# Causes, trends and severity of musculoskeletal injuries in Ghana

**DOI:** 10.1186/s12891-017-1709-8

**Published:** 2017-08-11

**Authors:** Eric Lawer Torgbenu, Emmanuel K. Nakua, Henry Kyei, Eric Badu, Maxwell Peprah Opoku

**Affiliations:** 1 Department of Physiotherapy and Rehabilitation Sciences, University of Health and Allied Sciences, School of Allied Health Sciences, Ho, Ghana; 2 Department of Community Health, Kwame Nkrumah University of Science and Technology, School of Medical Sciences, Kumasi, Ghana; 3 Department of Physiotherapy, Kibi Government Hospital, Kibi, Ghana; 4 Faculty of Education, University of Tasmania, Tasmania, Australia

**Keywords:** Musculoskeletal *injury*, *Rehabilitation*, *Hospital*, *Orthopaedic*, *Ghana*

## Abstract

**Background:**

Musculoskeletal [MSK] injuries are common causes of long-term pain and physical disability which affect many people worldwide. The economic and social impacts on the individual, society and national health systems are enormous making a matter of public health concern. Therefore, this study examined the causes and extent of MSK injuries in a referral hospital in Ghana.

**Methods:**

A prospective study design with consecutive sampling method was used to recruit patients admitted at Trauma Unit as well as those receiving orthopaedic reviews at St. Joseph’s Orthopaedic Hospital over a ten-month period. A structured questionnaire, Visual Analogue Scale (VAS) and Abbreviated Injury Scale (AIS) were used to collect data which were analysed descriptively using SPSS version 20.

**Results:**

A total of 269 MSK injury patients were identified - of these, 137 (50.9%) males with an average age of 38 years (SD = 19.88). Nearly half (49.1%) of the injuries sustained were fractures, and common causes were vehicular crash 113 (42.0%) and fall 68 (25.3%). Body parts affected most were the knee (19.62) and the mean levels of pain for all injuries were 6.04 ± 2.44 and 3.25 (±1.50) respectively.

**Conclusion:**

Ghana needs a healthy population to steer its development trajectory. Policy makers in Ghana should pay attention to both preventive as well as management of MSK injuries, or else, most of the country’s working class could live with lasting effects of injuries which may have significant impacts on the economy.

## Background

Musculoskeletal [MSK] injuries are the commonest causes of severe long-term pain and physical disability affecting hundreds of millions of people around the world [1]. MSK injury is known to lead to morbidity and mortality in Low and Middle-Income Countries [LMICs] [2]. According to the World Health Organization [WHO] in 2000, various kinds of injuries resulted in the death of 5.1 million people and accounted for 12% of the disability-adjusted life years (DALYS) worldwide which is expected to increase to 20% by the year 2020 [3–5]. Additionally, one-third of all health-related absence from work in developed countries are attributed to MSK conditions - a situation which is likely to be double in LMICs where a huge number of people suffer from non-fatalities every day [5]. This reinforces the need for attention to be given to trends in MSK injuries in LMICs where there seems to be numerous problems facing the healthcare system.

MSK injuries are among the most incapacitating in the adult population than any other group of disorders [6]. In the developed countries, like Canada, USA and Western Europe, the prevalence of physical disabilities caused by MSK condition has been estimated at 4–5% of the adult population [7]. The prevalence is higher among women and increases markedly with age [6]. Similarly, MSK injury is associated with 40% of chronic conditions; 54% of all long-term disability and 24% of all restricted activity days [6]. The pain and disability brought about by MSK conditions affect social functioning and mental health, further diminishing the patient’s quality of life [6, 8]. Therefore, having baseline information on the prevalence of MSK injury could enable policy makers to channel resources in both prevention and intervention.

The burden of this condition is heavily borne by LMICs, where preventive strategies are often nonexistent, untimely and even difficult to find [4]. It is important to reiterate the fact that the economic and social impact of MSK injuries on the individual, society, and the national health care systems is enormous [1–3]. Thus, in the absence of data on common trend in MSK injury, it will be difficult for healthcare system to put in place effective preventive and management strategies. It will be interesting to note that road traffic crashes have been identified as one of the major causes of serious MSK injuries in LMICs [2, 9, 10]. Also, they are one of the main causes of years lived with disability in all continents and major economies [6]. As fast cars are being developed and LMICs lacking the financial capacity to expand their roads but continue to use single lanes roads, road traffic crashes are bound to increase [4, 5]. These claims are unverifiable in the Ghanaian context; though road traffic crashes appear to be major national concern because of the number of people lost annually.

While there is considerable funding for control of communicable diseases, there has been little attention paid to either the documentation, prevention or the treatment of MSK problems in LMICs [9–12]. Despite the increasing burden of MSK disorders in LMICs, enforcement of policies and intervention have just begun to address injury control [13]. This lack of programmatic and policy drive towards the management of injuries is in part due to a lack of population-based and national research findings of MSK injuries. Apparently, availability of information in this regard may influence health decision-makers to allocate enough resources into injury prevention and management [2, 4, 5]. Thus, addressing the burden of injury in LMICs should be a public health priority where basic services that are low-cost, high-yield and appropriate, have to be made available in almost every health facility.

It is essential to mention that between 2000 and 2010, WHO launched the “Bone & Joint Decade” to raise awareness of the impacts of MSK injuries and disorders [12–14]. After more than 6 years of the campaign, the causes of MSK injuries affecting Ghanaians are yet to be documented as the country continue to rely on estimate given by WHO. This seems to suggest that the causes and burden of MSK injuries have not received scholarly attention in Ghana. Therefore, this study aimed to outline the etiological spectrum, injury patterns and short-term outcome of these injuries. The objectives of the current study included: 1) assessing the characteristics of injured patients; 2) the type of injury, causes and body parts affected; and 3) the severity of the injuries. It is anticipated that findings from this study could help raise awareness so that MSK disorders receive primacy in health strategy, training, research and management among clinicians and health policy makers.

## Methods

### Study design and setting

The study was a prospective design which involved MSK injury patients receiving orthopaedic care at St. Joseph’s Orthopaedic Hospital, Koforidua-Ghana, which serves a population of around 250,000. It is a Catholic Mission Hospital which aims to provide efficient and affordable speciality care in orthopaedic trauma. With approximately 180 beds, St. Joseph’s is among the few orthopaedic hospitals in Ghana with a catchment area encompassing the entire West African Sub-region. Annually, the hospital attends to over 40,000 cases with at least 11%, coming from outside the Eastern region where the hospital is located. Specifically, orthopaedic trauma cases constitute over 60% of the patients treated in the facility. The study was conducted from November 2013 to April 2014.

### Sampling technique

Consecutive sampling technique was used to recruit participants for this study. According to Lunsford and Lunsford, in consecutive sampling, only the available population is studied which makes it possible to have a good representation of the target population [15]. Patients were included if they presented with a MSK condition that was treated at the hospital. Researchers included all accessible patients with MSK conditions over a 6 month period who met the inclusion criteria, as and when they arrived at the hospital. The inclusion criteria were; 1) patients admitted at the Trauma Unit and 2) patients receiving orthopaedic reviews at the St. Joseph’s Orthopaedic Hospital. Conversely, patients were excluded if they: 1) absconded from the hospital, 2) were discharged against medical advice, 3) had other neurological conditions which may require other neurological investigations and management or 4) died before the assessment was completed.

### Method and process of data collection

After the necessary approval was granted, health professionals at the departments and wards were informed about the study. Because the hospital is a referral centre for orthopaedic trauma, the administrators informed us to collect data on Thursdays which is the day most patients went for consultation and review. In the discharge of their duties, the health professionals informed patients about the study. They explain the objectives of the study to patients and those who agreed to take part in the study were referred to the trained research assistant who was hired to collect data. Due to proximity of the facility to the researchers, the research assistant who was resident in the area was tasked to do the data collection. Some participants completed the questionnaire themselves while those who were unable to read were helped by the research assistant. Each participant spent an approximate time of 45 min – 1 h.

The questionnaire used in the data collection was developed from a review of literature. Information collected included demographic characteristics of participants, the diagnosis, injury causes, various parts of the body involved, the length of hospital stay and severity of injuries. Also, pain was measured with Visual Analogue Scale (VAS) while the severity of injury of the patient was assessed with Abbreviated Injury Scale (AIS). The VAS consists of a horizontal or vertical line exactly 10 cm equal parts with anchors at either end, i.e. 0 and 10. On the scale, 0 = no pain, 1–3 = mild pain, 4–6 = moderate and 7–10 = severe pain [15, 16]. Also, AIS is coded from a score of zero to six with a score of 0 = no injury, 1 = minor, 2 = moderate, 3 = serious, 4 = severe, 5 = critical and 6 = maximum [16].

### Data management and analysis

Data collected were entered into a computer and analysed using SPSS software version 20.0 with the help of a medical statistician. Data were summarised in the form of proportions and frequency tables for categorical variables. Means, median, mode and standard deviation were also used to summarise continuous variables. Means and standard deviations were illustrated using tables to present the causes of MSK injuries, pain levels and severity.

### Ethical consideration

The study was reviewed and approved by the Kwame Nkrumah University of Science and Technology (KNUST) /Komfo Anokye Teaching Hospital (KATH) Committee on Human Research, Publications and Ethics (CHRPE). Administrative approval was obtained from St. Joseph’s Orthopaedic Hospital, Koforidua. The confidentiality of the information provided by patients was assured. Patients were assured that refusal to participate in the study would not affect their treatment in the hospital. The study included only participants who gave their consent by signing the consent form and were informed that participation was voluntary. An information leaflet was given to the patients to read before consenting. The participants were informed that they could opt out of the study at any time and would not be required to give any reason for doing so.

## Results

### Demographic characteristics of participants

The demographic characteristics of the participants are summarized in Table 1. A total of 320 questionnaires were administered by the research assistant to the participants, of which 269 were fully completed and deemed valid for analyses of the study. Male participants constituted 137 (50.9%) while female patients were 132 (49.1%). The ages of the respondents ranged between 1 and 82 years with a mean of 38.0 years (SD 19.9 years). The majority of the participants were between the ages of 40 and 60 years (36.8%) while less than 5% were below the age of five. The majority of participants were married (51.7%), with 37.2% who were never married.

**Table 1:** Demographic characteristics of injury patients

Variables (*N* = 269)	Frequency	Percent
Age group		
< 5	13	4.8
5 - <19	34	12.6
19 - <25	32	11.9
25 - <40	57	21.2
40–60	99	36.8
> 60	34	12.6
Mean Age (SD)	37.96 (19.88)	
Sex		
Male	137	50.9
Female	132	49.1
Category		
Adult	222	82.5
Children	47	17.5
Marital status		
Single	100	37.2
Married	139	51.7
Separated	4	1.5
Divorced	18	6.7
Widowed	8	3.0

### Distribution of diagnoses

Table 2 shows percentage distribution of participants regarding age, gender and marital status based on their respective diagnoses. One hundred and thirty-two (49.1%) of participants presented fracture cases, 32 (11.9%) were arthritis, 27 (10.0%) had dislocation, 40 (14.9%) had back pain, 18 (6.7%) were of ligament injury, 6 (2.2%) were amputation while 14 (5.2%) were tendon injury. Seventy-nine out of the 132 participants diagnosed with fracture were male while only four were amputees. On the other hand, 53 of the 132 fracture patients were females compared to only two who were amputees Regarding age, over 30% were between 40 and 60 years while less than 5% were less than 5 years. Out of the 132 fracture patients, 51 were between 40 and 60 years while only two in the same age bracket were amputated. Of those less than 5 years of age, 12 were diagnosed with ligament injuries while only one was having a tendon injury. On marital status, over 50% of participants were married compared to less than 2% who were separated. Out of the total number of participants diagnosed with fracture (132), 53% were married while less than 2% each were separated and widowed. Of those who were amputated, all were married.

**Table 2:** Percentage distribution of characteristics of participants reporting pain or discomfort due to injury or crash with various diagnoses

Diagnosis								
Variables (*N* = 269)	Fracture (*n* = 132)	Arthritis (*n* = 32)	Dislocation (*n* = 27)	Back pain (*n* = 40)	Ligament injury (*n* = 18)	Amputation (*n* = 6)	Tendon injury (*n* = 14)	Total (%)
Males	79 (59.8%)	6 (18.8%)	13 (48.1%)	21 (52.5%)	8 (44.4%)	4(66.7%)	6(42.9%)	137 (50.9%)
Females	53 (40.2%)	26 (81.3%)	14 (51.9%)	19 (47.5%)	10 (55.6%)	2(33.3%)	8(57.1%)	132 (49.0%)
Age								
< 5	-	-	-	-	12(66.7)	-	1(7.1)	13 (4.83)
5–19	18 (13.6)	2 (6.25)	7 (25.9)	4 (10.0)	3 (16.7)	-	-	34 (12.6)
19–25	13 (9.8)	1 (3.1)	5 (18.5)	10 (25.0)	3 (16.7)	-	-	32 (11.9)
25–40	34 (25.8)	9 (28.1)	3(11.1)	9 (22.5)	-	2	-	57 (21.2)
40–60	51 (38.6)	16(50.0)	8 (29.6)	13 (32.5)	-	2	9(64.3)	99 (36.8)
> 60	16 (12.1)	4 (12.5)	4 (14.8)	4 (10.0)	-	2	4(28.6)	34 (12.6)
Marital status (%)								
Single	48 (36.4)	6 (18.8)	13 (48.1)	14 (35.0)	18 (100.0)	-	1 (7.1)	100 (37.2)
Married	70 (53.0)	20 (62.5)	10 (37.0)	26 (65.0)	-	6 (100.0)	7 (50.0)	139 (51.7)
Separated	2 (1.5)	-	-	-	-	-	2 (14.3)	4 (1.5)
Divorced	10 (7.6)	4 (12.5)	2 (7.4)	-	-	-	2 (14.3)	18 (6.7)
Widowed	2 (1.5)	2 (6.3)	2 (7.4)	-	-	-	2 (14.3)	8 (3.0)

### Body parts affected, causes and severity of injury

Most injuries were related to the knee (19.6%), followed by the low back (14.6%), thigh (11.4%), ankle (10.8%) and shoulder (10.1%). The neck recorded the least injuries (0.6%). Few injuries occurred at the foot, upper back and the thumb/finger regions. Table 3 presents the distribution of causes of injuries among participants. Vehicular crash was found to be the highest cause of MSK injuries, representing 42.0% of injury causes, while assault was 0.7%. Out of 132 participants diagnosed of fracture, 90 were due to vehicular crash while only two indicated others. About arthritis (32), 22(68.8%) indicated other while only two mentioned assault. In relation to dislocation (27), 15(55.6%) indicated vehicular crashes while 12 mentioned fall. For back pain (40), 26(65%) indicated other causes while only two indicated gunshot as cause of their condition. For amputation, four indicated other while two indicated vehicular crash as a cause of their condition.

**Table 3:** A distribution of the causes of the various musculoskeletal injuries as diagnosed

Diagnosis							
Cause of injury (*N* = 269)	Fracture (*n* = 132)	Arthritis (*n* = 32)	Dislocation (*n* = 27)	Back pain (*n* = 40)	Ligament injury (*n* = 18)	Amputation (*n* = 6)	Tendon injury (*n* = 14)
Fall (*n* = 68)	36(27.3)	4(12.5)	12(44.4)	12(30)	-	-	4(28.6)
Assault (*n* = 2)	-	2(6.3)	-	-	-	-	-
Vehicular crash (*n* = 113)	90(68.2)	4(12.5)	15(55.6)	-	-	2(33.3)	2(14.3)
Gunshot (*n* = 6)	4(3.0)	-	-	2(5)	-	-	-
Others (*n* = 80)	2(1.5)	22(68.8)	-	26(65)	18(100)	4(66.7)	8(57.1)

### Level of pain and injury severity

Table 4 presents the distribution of injury severity recorded on the AIS. Participants were asked to circle the number that best explain their condition. In all, it was found that 74 participants (27.5%) indicated that their conditions were serious while only two (0.7%) were in critical conditions. When it comes to individual diagnoses, out of 132 participants diagnosed with a fracture, over 90 had serious and severe conditions. On arthritis, 18 had no injury while only two participants indicated moderate injuries. On dislocation, out of 27 participants, 16 (21.6%) reported serious conditions compared to three (9.7%) indicated moderate conditions. On back pain, out of 40 participants diagnosed, 20 (37.0%) were without injury while only two were in critical condition. In terms of ligament injury (18), eight each indicated no injury and serious injury respectively (see Table 4 for details).

**Table 4:** Distribution of injury severity indicate on the AIS

*N* = 269	No injury *n* = 54 (20.0%)	Minor *n* = 40 (14.9%)	Moderate *n* = 31 (11.5%)	Serious *n* = 74 (27.5%)	Severe *n* = 68 (25.2%)	Critical *n* = 2 (0.7%)
Fracture (*n* = 132)	-	14(35.0)	26(83.9)	40(54.1)	52(76.5)	-
Arthritis (*n* = 32)	18(33.3)	6(15)	2(6.5)	-	6(8.8)	-
Dislocation (*n* = 27)	-	4(10.0)	3(9.7)	16(21.6)	4(5.9)	-
Back pain (*n* = 40)	20(37.0)	12(30)	-	6(8.1)	-	2(100)
Ligament injury (*n* = 18)	8(14.8)	-	-	8(10.8)	2(2.9)	-
Amputation (*n* = 6)	-	-	-	4(5.4)	2(2.9)	-
Tendon injury (*n* = 14)	8(14.8)	4(10.0)	-	-	2(2.9)	-

The mean score of both pain and injury severity were calculated (see Table 5 for details). The overall mean level of pain as reported by the participants was 6.04 ± 2.44. Dislocation recorded the highest mean of 6.85(±2.05) while tendon injury recorded the lowest mean of 4.00(±3.37). In addition, fracture and amputation recorded a mean of 6.38(±2.18) and 6.00(±2.37) respectively. The overall mean injury severity level reported by participants using the AIS was 3.25(±1.50). Amputation recorded the highest mean severity level of 4.33 (±0.52) while tendon injury recorded the least mean of 3.25 (±1.50). In the same way, fracture and dislocation recorded an average of at least three on the scale.

**Table 5:** Distribution of the mean levels of pain

Diagnosis	Average Level of pain (VAS)	Injury severity (AIS)
Fracture	6.38 (±2.18)	3.98 (±1.01)
Arthritis	5.81 (±2.69)	2.12 (±1.52)
Dislocation	6.85 (±2.05)	3.74 (±0.90)
Back pain	5.31 (±2.21)	1.89 (±1.39)
Ligament Injury	4.00 (±3.33)	2.78 (±1.67)
Amputation	6.00 (±2.37)	4.33 (±0.52)
Tendon Injury	5.21 (±2.23)	1.86 (±1.41)
Total	6.04 (±2.44)	3.25 (±1.50)

Mean (Standard Deviation)

## Discussion

This study aimed to document the extent and causes of MSK injuries in a major referral hospital in Ghana. As found by the study, the leading cause of MSK injury in this study was vehicular crash. This finding is not different from studies in other countries where the vehicular crash was reported as a principal cause of MSK injuries [4, 9, 12, 17, 18]. The contributing rise of vehicular crash in Ghana may be due to second-hand vehicles, bad roads and weak enforcement of traffic laws (5). Perhaps, both drivers and pedestrians lack understanding and interpretation of road signs which could be responsible for the increasing number of vehicular crashes. Also, falls were recorded as the second leading cause of MSK injuries among participants in this study. This finding corroborates a study by Onwudike, Olaloye and Oni [19] who identified falls, assaults and gunshots as among the leading causes of injuries. This opens the debate on the extent of consideration given to the needs of diverse individuals during building and infrastructural development. Notwithstanding, the ability of individuals who suffered injuries to adapt to the already built physical environments is an issue which is gaining gradual discussions in Ghana as existing empirical research has found the physical environments to be unfriendly [20]. In the interest of all persons, it is vital for builders to make provision that could guarantee the safety of users of the facilities.

The study identified fracture, arthritis, dislocation, back pain, amputation, ligament and tendon injuries as the main MSK injuries presented by participants. Fracture cases were the most reported injury by the participants in this study. It was confirmed by the findings by Lee & Porter [1] that fractures were the commonest condition reported in their study. This might have had impact on the daily living conditions of individuals as they may be unable to perform their duties as expected. For instance, Järvinen, Järvinen and Kaariainen [21] identified MSK injuries to be common in professional and amateur athletes. Payne, Kinmont and Moalypour [22] mentioned that any injury to a body part also causes traumatic injury to both bone and surrounding soft tissue. Their study also revealed that 90% of muscle and tendon injuries are usually caused by excessive strain which may result in inability of the individual to train or compete for several weeks. These injuries have high tendencies to recur which might account for many chronic MSK conditions. Thus, the difficulty in tackling such injuries presents an urgent concern for LMIC where not much investment are provided for the development of healthcare system.

Back pain was also one of the leading conditions participants reported. Participants who reported having back pain attributed the condition to other causes rather than vehicular crash, falls, assaults and gunshot in this study. This confirmed the findings by Waddell et al. [23] that it is often difficult to associate back pain with some of the causes of MSK injuries. Although the prevalence of back pain varied from population to population, a study by Brage and Laerum [24] revealed that 60–80% of world’s population experience recurring back pain and hence, it was not surprising that participants indicated other causes. Potentially, there are daily habits such as sitting, carrying or other tasks that could cause back pain. Perhaps, individuals do not get education in some daily life activities which might have impact on their lives.

The results of this study revealed that conditions presented at the hospital were of different severity scores. For instance, participants diagnosed of back pain and tendon injuries reported low scores on the injury scale. Although this study did not measure disability levels, it somehow contradicts a study by Borenstein [17] who found back pain as a leading cause of disability and activity limitation. Notwithstanding, this study found it to be one of the most reported complaints of participants and hence, further study could establish its relationship with disability. Additionally, participants with fractures, dislocation, amputation and ligament injuries recorded the highest severity score which means that they might be experiencing, or be at risk of, disability. Specifically, participants who had fractures recorded one of the highest scores on the severity scale. They also form the majority of participants recruited in this study. It is possible that there is either lack of trained personnel or lack of infrastructure to properly handle fracture conditions. It is worth mentioning that fractures may recur if not properly managed which might negatively affect patients. Hence, best intervention strategies may be needed to enable individuals to get back to the society. Unsurprisingly, amputation was found to record the highest severity. This is likely to leave individuals with permanent disability and participants who had amputation would need other assistance to be physically functional again. This study is also in agreement with Woolf and Pfleger’s [8] findings that amputation is the commonest cause of severe long-term and physical disability.

It is imperative to mention that most of the participants diagnosed were found to be between the ages of 40–60 years which seems to be critical period where people are mainly engaged in productive activities. Within this period, people are expected to stay healthy to be able to meet the demands of life. However, with intense working conditions and possibly the desire to excel in one’s field of endeavour, it is possible that individuals may be unable to regularly exercise or eat healthy diets making them susceptible to injuries that might keep them out of the world of work. A healthy population is a wealthy population [25] and therefore, injuries need to be handled well in order not to burden society with individuals who would continue to depend on others for survival. This finding is consistent with studies by Chang et al. [26] and Rubenstein [27] who mentioned the risk of injuries among older people. Although these studies were conducted with participants aged 60 years and over, it could somehow be compared to the Ghanaian context where the average life expectancy is a little over 50 years. Healthy living appears to be an important public health concern for countries with low mortality rate. Ghana needs to work on this area in order to possibly, prolong the life expectancy of its citizens.

The levels of pain indicated that participants diagnosed with fracture, dislocation and amputation experienced the most pain on the VAS. This is possibly a major wake-up call for health professionals, especially those involved in rehabilitation, to critically evaluate their approach to disabling conditions and adopt mechanisms which they could use to handle patients. There is the need for individuals to get back into the community to continue working or live independently like any other person within the society. Therefore, it is essential for health professionals to acquire the best training as well as resourced to rehabilitate patients back into the society.

### Implication for policymaking

The study has implications for policy making in Ghana. The study revealed that a major cause of MSK injuries is vehicular crash. This calls for intensified campaigns, workshops and seminars geared towards defensive driving skills and the use of road signs which could help reduce the number of vehicular crashes on the roads. Elvik et al., [4] in a study in Europe reports that the high rate of road crashes and traffic injuries, especially among company drivers, led the companies to adopt campaigns such as educating their employees against the use of attention-demanding activities such as using mobile phones while driving as well as organizing workshops to educate them on the need to drive responsibly. In Ghana, road traffic controllers must ensure that careless drivers are dealt with according to the traffic rules and regulations. Law enforcement agencies such as the Motor Traffic and Transport Unit [MTTU] of the Ghana Police Service as well as National Road Safety Commission [NRSC] must intensify rules and regulations pertaining especially to our roads to reduce the increasing number of vehicular crashes in the country.

Secondly, apart from road crashes, there were other factors found to cause injuries among participants. It is recommended that educative, interventionist programmes geared towards prevention and management of injuries are emphasised. For instance, Chang et al., [26] and Rubenstein [27] suggested the essence of regular exercises which could help reduce the high rate of injuries caused by falls among older persons. Ghana Health Service and Ghana Physiotherapy Association [GPA] could lead this campaign. Also, Mock and Cheridan [9] mentioned that the increase in the number of ambulance units across Mexico enabled rapid response to victims of vehicular crashes and shooting incidents. This could be adopted in Ghana to enable early response to road crashes. Also, it is prudent that more health workers, especially nurses and emergency attendants, should be trained in management and emergency handling of MSK injuries. Furthermore, rehabilitation centres should be instituted, or the existing facilities should be resourced to assist individuals who become victims of MSK injuries. This will help with the management of most of the chronic injuries and the rehabilitation of the disabling conditions.

## Conclusion

The study aimed to describe the trends, causes and severity of MSK injuries in Ghana**.** The study found the knee as the most reported body part with the neck being the least. Vehicular crashes and falls were the most prevalent causes of MSK injuries. Fractures, Arthritis and dislocation were the major conditions seen across age groups among the study participants. Participants with dislocation reported the highest mean pain levels which were closely followed by participants diagnosed with fracture. Individuals who suffered back pain reported the lowest pain thresholds. Ghana needs a healthy population to steer its development trajectory. Policy makers in Ghana should pay attention to both preventive as well as management of MSK injuries, or else, most of the country’s working class could live with lasting effects of injuries which may have significant impacts on the economy. In order for the country to help manage MSK injuries, it is important that measures such as investment in resources and quality of training of specialists be implemented to help address this menace. Also, rehabilitation centres need to be established to help in restoring victims of MSK injuries to their productive lives.

The results have to be interpreted with caution due to the limited scope of the study. Participants were only recruited from one major hospital in Ghana which means that the results may not be representative of all patients who have been diagnosed with MSK injuries. Also, it is important to mention that participants who absconded from treatment were not included in the final reporting of the study. Thus, it is important that future studies be conducted in other hospitals and rehabilitation centres so as to have a holistic understanding of MSK injuries. Despite these limitations, the study has provided a descriptive picture of the causes and severity of MSK conditions that has received little scholarly attention in Ghana.

## Abbreviations

AIS: Abbreviated Injury Scale
DALYS: Disability-adjusted life years
GPA: Ghana Physiotherapy Association
HIV: Human Immunodeficiency Virus
LMIC: Low and Middle Income countries
MSK: Musculoskeletal
MTTU: Motor Traffic and Transport Unit
NRSC: National Road Safety Commission
SD: Standard deviation
VAS: Visual Analogue Scale
WHO: World Health Organization
